# Magnetic resonance imaging biomarkers in hepatocellular carcinoma: association with response and circulating biomarkers after sunitinib therapy

**DOI:** 10.1186/1756-8722-6-51

**Published:** 2013-07-10

**Authors:** Dushyant V Sahani, Tao Jiang, Koichi Hayano, Dan G Duda, Onofrio A Catalano, Marek Ancukiewicz, Rakesh K Jain, Andrew X Zhu

**Affiliations:** 1 Division of Abdominal Imaging and Intervention, Harvard Medical School and Massachusetts General Hospital, 55 Fruit Street, White 270, Boston, MA 02114, USA; 2 Department of Radiology, ChangZheng Hospital Affiliated to Second Military Medical University, 415 Fengyang Road, Shanghai 200003, China; 3 Steele Laboratory, Massachusetts General Hospital, 100 Blossom Street, Cox-734, Boston, MA 02114, USA; 4 Department of Radiology, SDN Foundation, IRCCS 80143, Naples, Italy; 5 Cancer Center, Massachusetts General Hospital, 55 Fruit Street, LH/POB 232, Boston, MA 02114, USA

**Keywords:** Hepatocellular carcinoma, Antiangiogenic treatment, Image biomarker, Dynamic contrast-enhanced MRI, Diffusion-weighted imaging, Circulating biomarker

## Abstract

**Background:**

To investigate the hypothesis that MRI derived diffusion-weighted imaging (DWI) and perfusion (MRP) parameters are sensitive image biomarkers for monitoring early antiangiogenic effects and predicting progression free survival (PFS) in advanced hepatocellular carcinoma (HCC).

**Methods:**

In this phase II clinical trial, 23 of 34 patients were included in the imaging and circulating biomarker study. DWI and MRP were performed at the baseline and at 2-weeks after initiation of sunitinib. The imaging protocol included an axial DWI sequence using b values of 50, 400 and 800 sec/mm^2^, and MRP using a series of coronal 3D-VIBE following 20 ml of Gd-DTPA at 2 ml/sec. These parameters were compared with clinical outcome and PFS at 6-months. Correlation between changes in MRI parameters and plasma biomarkers was also evaluated.

**Results:**

After 2-week of sunitinib, substantial Ktrans changes in HCC were observed from median baseline value 2.15 min^−1^ to 0.94 min^−1^ (*P* = 0.0001) with increases in median apparent diffusion coefficient (ADC) from 0.88 × 10^-3^ mm^2^/s to 0.98 × 10^-3^ mm^2^/s (*P* = 0.0001). Tumor size remained unchanged by RECIST and mRECIST (both *P* > 0.05). Patients who showed larger drop in Ktrans and Kep at 2 weeks correlated with favorable clinical outcome, and higher baseline Ktrans and larger drop in EVF correlated with longer PFS (all *P* < 0.05). There was a significant association between a decrease in sVEGFR2 and the drop in Ktrans and Kep (*P* = 0.044, *P* = 0.030), and a significant and borderline association between decrease in TNF-α and the drop in Ktrans and Kep, respectively (*P* = 0.051, *P* = 0.035).

**Conclusion:**

In HCC, MRP may be a more sensitive biomarker in predicting early response and PFS following sunitinib than RECIST and mRECIST.

**Trial registration:**

ClinicalTrials.gov: NCT00361309

## Background

Hepatocellular carcinoma (HCC) is the sixth most common cancer and the third most common cause of cancer-related death worldwide and is responsible for more than 500,000 deaths every year globally [1]. Advanced HCC carries a poor prognosis, and systemic therapy with cytotoxic agents provides marginal benefit [2,3]. Over the past few years, significant progress has been made in our understanding of the molecular pathogenesis of HCC, which led to the rationale for the use of novel targeted agents in clinical trials. Due to highly angiogenic microenvironment in HCC, antiangiogenic agents such as sorafenib, bevacizumab and cediranib have been tested in clinical trials for the treatment of HCC [4-10]. While sorafenib has shown increased survival in HCC, other anti-VEGF agents have shown mixed results [11]. Sunitinib (Sutent; Pfizer, New York, NY) is an oral multitargeted receptor tyrosine kinase inhibitor (TKI), which has been approved for the treatment of renal cell carcinoma, imatinib-resistant gastrointestinal stromal and pancreatic neuroendocrine tumors [12]. Although sunitinib demonstrated early evidence of modest antitumor activity in advanced HCC patients from single arm phase II studies [13], a randomized phase III trial failed to demonstrate either superiority or non-inferiority of sunitinib when compared with sorafenib. This clinical experience indicates that only some patients benefit from these targeted therapies. The mechanisms of action in targeted agents, which often cause cytostatic effect rather than cytotoxic effect, are different from conventional chemotherapy, and therefore, the response assessment criteria currently in use might be inadequate. Imaging modalities such as computed tomography (CT) and magnetic resonance imaging (MRI) are commonly used in phases II and III clinical trials. They provide reliable and reproducible anatomical assessment of changes in tumor size. However, antiangiogenic therapies for HCC are known to induce tumor necrosis and may cause no-change or relative enlargement of the tumor size on imaging thereby leading to inappropriate categorization of an otherwise responsive disease, as stable or progressive based on established methods such as Response Evaluation Criteria In Solid Tumors (RECIST) [14]. Therefore, the European Association for the Study of the Liver (EASL) guidelines recommended that the response criteria be amended to take into account viable tumor (contrast enhancement in the arterial phase) [15], and the American Association for the Study of Liver Disease (AASLD) developed a set of guidelines that included a formal modification of the response assessment based on the RECIST criteria and aimed to translate into the concept of viable tumor, which are referred to as modified RECIST (mRECIST) criteria [16].

With the advancements in MR technology and availability of commercial software, MR perfusion (MRP) and diffusion-weighted imaging (DWI) have found their applications in HCC [17]. DWI uses phase-defocusing and phase-refocusing gradients, which allow evaluation of the rate of microscopic water diffusion as a marker of cellular density and integrity. Using a dynamic MRI, hemodynamic parameters for permeability measurement, such as transfer constant (Ktrans), redistribution rate constant (Kep) and extracellular volume fraction (EVF), can be quantitated [18]. Several studies had attempted to assess if DWI and MRP derived tumor parameters could be used for assessment of response to therapies [12,19-21]. Therefore, we had formulated and investigated the hypothesis that the DWI or MRP derived tumor parameters are more sensitive image biomarkers when compared with tumor burden measurements as defined by RECIST or mRECIST in a clinical trial of sunitinib for monitoring early antiangiogenic effects and predicting progression free survival (PFS) in advanced HCC. We also postulated that imaging biomarkers correlate with circulating biomarkers measured in plasma. Additional objective was to compare the DWI and MRP parameters of tumor thrombus and their changes in patients with different clinical outcome and PFS.

## Methods

### Patients

The protocol for this phase II clinical trial was in compliance with Health Insurance Portability and Accountability Act (HIPAA) Regulations and was approved by the The Institutional Review Board (IRB) at Dana Farber Harvard Cancer Center. All patients were required to provide written informed consent before study participation according to institutional and federal guidelines. Eligibility criteria included histologically proven, measurable, locally advanced, recurrent or metastatic HCC; no more than one prior systemic regimen; prior chemoembolization therapy only if performed more than 4 weeks before study entry and measurable disease present outside of the chemoembolization field; age 18 years; Eastern Cooperative Oncology Group performance status of 0 or 1; Cancer of the Liver Italian Program (CLIP) score 0–3; and adequate hepatic, renal, and bone marrow function. Exclusion criteria included concurrent malignancies; significant medical comorbidities; significant cardiovascular disease including uncontrolled hypertension, myocardial infarction, and unstable angina; New York Heart Association grade 2 or greater congestive heart failure; prolongation of QTc of more than 450 msec in screening ECG or history of familial long QT syndrome; history of bleeding; proteinuria at baseline (more than 2 g/d); pregnancy or lactation; central nervous system metastases; or an inability to provide written informed consent. Thirty-four patients with advanced HCC were enrolled and 23 were included in the current study. The prospective study cohort included 7 men and 16 women (age range, 38 ~ 76 years; median age, 62.6 years).

### Antiangiogenic treatment

The eligibility, treatment schedule, and dose modification schema have been detailed previously [10]. Briefly, eligible patients received sunitinib at a dose of 37.5 mg daily by mouth for 28 days followed by 14 days of rest in 6-week cycles. Patients with grade 3 or 4 toxicities underwent dose reduction to 25 or 12.5 mg daily, respectively. Treatment was continued until progression, unacceptable toxicity, or withdrawal of consent. Response and progression were evaluated using the RECIST after completion two cycles of sunitinib therapy.

### Imaging protocol

This clinical trial was designed not only to investigate the role of DWI and MRP for monitoring early antiangiogenic treatment effects but also to study the overall survival and PFS. The DWI, MRP and delayed postcontrast T1-weighted images were performed at base line and two weeks after initiation of antiangiogenic treatment. A restaging contrast-enhanced MRI (CE-MRI) was performed at the end of cycle 2 treatment with sunitinib for response status and then at every 6-weeks until disease progression. The data acquisition parameters, the same injection protocol and the anatomic location for scanning, including the total duration, were kept constant for each patient and for each repeat DWI, MRP and CE-MRI study.

#### DWI

DWI of the liver was performed using a phased array body coil on a 1.5-T MRI system (Avento; Siemens, New York, NY) using the following protocol. T1-weighted in-phase and out- of-phase images (repetition time (TR)/ echo time (TE), 122 ~ 159/2.38 ~ 4.72 msec; one signal acquired; flip angle, 70 degree; 20 slices; section thickness, 5 ~ 7 mm; 1-mm interslice gap) and axial respiratory-triggered fast spin-echo T2-weighted images (TR/TE, 3500 ~ 4494/65 ~ 85 msec; one signal acquired; flip angle, 53 degree; echo train length, 15; 20 slices; section thickness, 5 ~ 8 mm; 1-mm interslice gap) were acquired first. Thereafter, an axial respiratory-gated echo-planar diffusion-weighted (DW) sequence with spectral fat saturation was performed by using the following parameters: b values of 50, 400 and 800 sec/mm^2^; TR/TE, 4959 ~ 7936/44 ~ 74 msec; two signals acquired; echo train length, 1; flip angle, 60 ~ 90 degree; 20 slices; section thickness, 5 ~ 8 mm; 1-mm interslice gap; field of view, 263 × 350; matrix, 144 × 192. The total acquisition time is 3 ~ 6 min.

#### MRP

MRP of the liver was performed using a phased array body coil on the same 1.5-T MRI system (Avento; Siemens, New York, NY) using the following protocol. At first, three dimensional volume interpolated excitation coronal T1 sequence (VIBE) was obtained in a breath hold before contrast media injection using the following parameters: TR = 5 msec, TE = 1.58 msec, 5-mm slice thickness, 1-mm interslice gap, 20 slices, 123 × 192 matrix, and field of view of 400 × 400 mm. Thereafter, through the 20-guage peripheral intravenous line in the arm, 0.1 mmol/kg body weight of gadopentetate dimeglumide contrast media (Magnevist; Berlex, Montville, NJ) was power injected at 2 mL/sec, followed by a saline chase of 20 mL at a rate of 2 mL/sec. Then, MRP acquisition was performed. A series of coronal T1-weighted three-dimensional volume interpolated excitation (VIBE) images were obtained after 5-second delay after the initiation of contrast media injection, and the scanning continued for up to 4 minutes and 40 seconds. The acquisition parameters included: TR = 5 msec, TE = 1.58 msec, 5-mm slice thickness, 0-mm interslice gap, 20 slices, 123 × 192 matrix, 15-degree flip angle, and field of view of 400 × 400 mm. Two consecutive 7-second acquisitions forming two different time points were repeated 10 times with a delay of 14 seconds between them. The scanning time in every acquisition was 14 seconds with a break of 14 seconds, and the patients were asked to hold their breath during acquisition. Finally, delayed postcontrast T1-weighted images were taken as follows: axial and coronal two-dimensional T1-weighted fat-saturated gradient echo (GRE) sequences using TR = 150 msec, TE = 2.1 msec, 160 × 256 matrix, 20 slices, 5-mm thickness, and 0-mm interslice gap. For the measurement of tumor burden and diameter of tumor thrombus in portal vein, postcontrast T1-weighted images were applied.

#### CE-MRI

CE-MRI of the liver was performed using a phased array body coil on the same 1.5-T MRI system (Avento; Siemens, New York, NY) using the following protocol. A total of 0.1 mmol/kg body weight of gadolinium-diethylenetriaminepentaacetic acid (Gd-DTPA) contrast media (Magnevist; Berlex, Montville, NJ) was power injected at 2 mL/sec, followed by a saline chase of 20 mL at a rate of 2 mL/sec. The arterial, portal and delayed phase scanning were performed after 35, 65 and 280 seconds from the initiation of contrast medium bolus. Three dimensional volume interpolated excitation axial T1 sequence was obtained in a breath hold after contrast media injection using the following parameters: TR = 5 msec, TE = 1.58 msec, 5-mm slice thickness, 0-mm interslice gap, 20 slices, 123 × 192 matrix, and field of view of 400 × 400 mm.

### Image processing

Data were processed at a picture archiving and communication system (PACS) (Impax 4.0; Agfa, Mortsel, Belgium) by two experienced radiologists with 13 and 10 years of experience in liver imaging. To obtain permeability maps, MRP images were processed at pixel resolution by using a commercially available full time point (fTP) model (iCAD Sciences, White Plains, NY) to analyze the time evolution of contrast enhancement.

Tumor size was measured in the longest cross-sectional dimension for each lesion based on RECIST 1.1 guidelines. And viable tumor diameter was measured in the longest cross-sectional dimension for enhancing component of each lesion based on mRECIST guidelines. The sum of the longest dimensions of selected target lesions in each patient was computed, and the absolute and percent changes of the sum from the baseline to post treatment evaluation were computed for each patient. Diagnostic standard and diameter measurement for tumor thrombus in portal vein was referred to the criteria described by Shah et al. [22].

The apparent diffusion coefficient (ADC) map was automatically generated on the imager console from the DWI sequence; the selected b values (50, 400 and 800 sec/mm^2^) were used for ADC quantification. For ADC quantitative analysis, the nonenhanced images showing the maximal diameter of the HCC and the maximal diameter of the tumor thrombus in the portal vein were respectively selected as reference [23]. Definitions of MRP parameters and the model used for generating functional maps were described as before [24,25]. Briefly, for each MRP acquisition, the fTP-pharmacokinetic image analysis platform implements the Tofts pharmacokinetic model to quantify vascular permeability (Ktrans, EVF and Kep).

On DWI and MRP postprocessed images, we manually drew region of interests (ROI) in all the anatomic locations from the section in which the tumor was first visible to the last section in which the tumor was visible to enable whole tumor evaluation, and corresponding values were acquired. ROI included at least two-thirds of the area of the HCC and at least three-fourths of the area of the thrombus in the portal vein. To minimize volume averaging, we enlarged the images and placed the ROI within the thrombus. Care was taken to avoid including in the ROI any area outside the HCC and the thrombus. For patients with multiple lesions, we drew ROI for all and computed a median value for analysis of parameters. Then, the absolute and percent changes in ADC value and MRP parameters from the baseline and after therapy were calculated for each patient.

### Circulating biomarkers

For measurement of angiogenic proteins and inflammatory cytokines in plasma, peripheral blood was obtained at baseline and 14 days after the first dose of sunitinib. All samples were collected in ethylenediaminetetraacetic acid (EDTA)-containing Vacutainers. Plasma analysis was carried out for circulating vascular endothelial growth factor (VEGF), placental-derived growth factor (PlGF), soluble VEGF receptor (VEGFR) 1, basic fibroblast growth factor (bFGF), interleukin (IL)-1β, IL-6, IL-8, and tumor necrosis factor α (TNF-α) using multiplex array plates from Meso-Scale Discovery (Gaithersburg, MD), and for soluble VEGFR2, soluble VEGFR3, stromal-derived factor 1α (SDF1α), VEGF-C, and soluble c-KIT from R&D Systems (Minneapolis, MN) [12]. Samples were run in duplicate.

### Statistical analysis

Data were analyzed using SPSS statistical software, version 10.0, SPSS Inc, Chicago, IL and R (R Foundation for Statistical Computing, Vienna, Austria). We present medians with ranges of various parameters of the HCC and tumor thrombus at baseline and following antiangiogenic treatment. Changes in ADC value and MRP parameters of HCC and tumor thrombus as well as tumor burden measures were expressed as percent changes and tested statistically using paired exact Wilcoxon signed rank test for comparison at the baseline and at the 2 weeks after initiation of treatment. For correlation with clinical outcome, patients were divided into two groups: those with progressive disease (PD) and those with either stable disease (SD) or a partial response (PR). Similarly, for correlation with PFS, patients were divided into two groups: those with PFS ≤ 6 months and those with PFS>6 months. The baseline values and percent changes in various parameters after sunitinib administration in these groups were compared using the two-sample exact Wilcoxon rank sum test. Correlation between changes in MRI parameters and plasma biomarkers at day 15 after sunitinib treatment in advanced HCC patients was performed using Kendall’s correlation coefficients. In this study, a *P-*value of less than or equal to 0.05 was considered to indicate a statistically significant difference.

## Results

The clinical outcome of patients from the phase II trial has been previously reported [10]. Briefly, of the 23 patients enrolled in the current study who were evaluable for efficacy after completion two cycles of sunitinib therapy, one (4.4%) had a confirmed PR and 15 (65.2%) had SD, the other 7 (30.4%) showed PD. In 14 patients (60.9%) PFS ≤ 6 months was encountered and the other 9 had PFS>6 months (39.1%). In addition, 9 of 23 patients (39.1%) were categorized as having tumor thrombosis; 5 (55.6%) showed PFS ≤ 6 months and the other 4 had PFS>6 months (44.4%).

### RECIST, mRECIST, DWI and MRP parameters at baseline and after 2-week of sunitinib therapy in HCC and tumor thrombus

**Figure 1:**
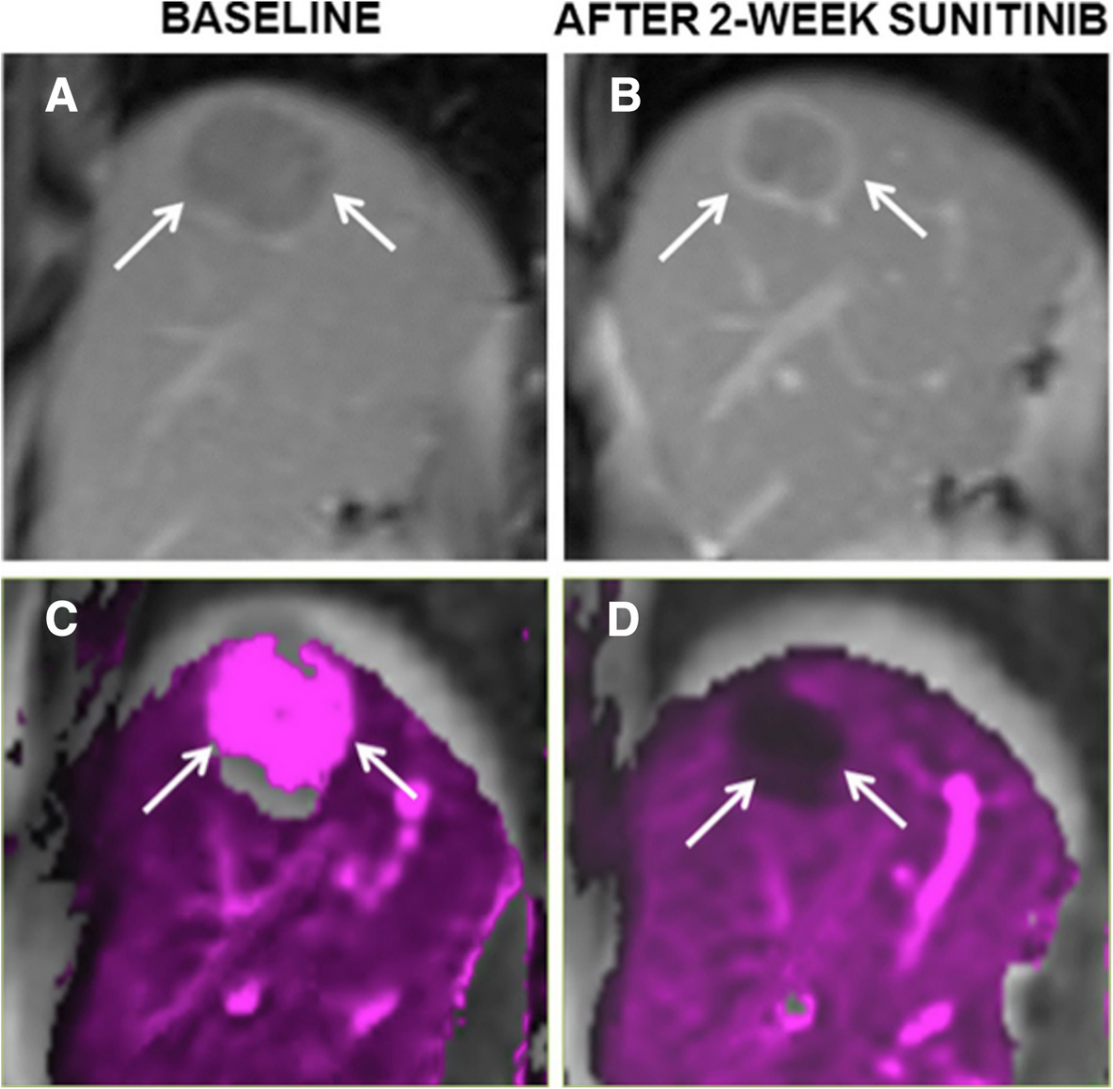
**Functional transfer constant map of HCC during sunitinib therapy.** Coronal enhanced MR T1-weighted images **(A**, **B)** and functional transfer constant (Ktrans) maps from MR perfusion **(C**, **D)** at baseline and after 2-week of sunitinib therapy in a 47-year-old man with HCC (arrows). After two weeks of treatment with sunitinib, HCC showed a 96% drop of Ktrans, which presented a conspicuity of permeability change.

**Figure 2:**
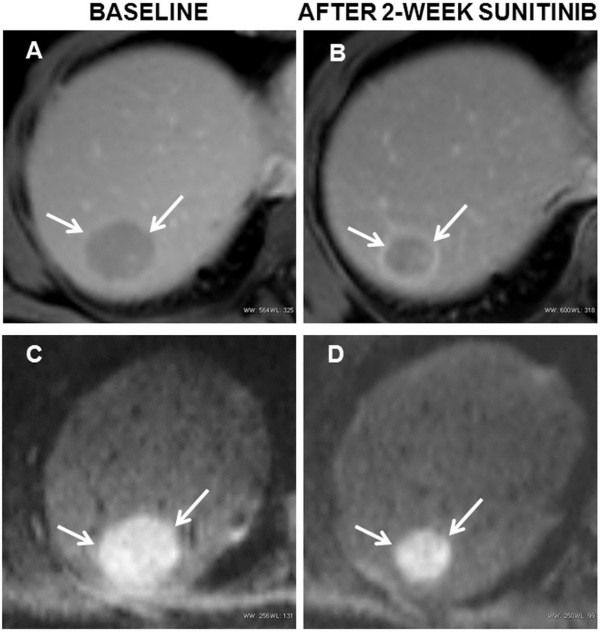
**MR diffusion weighted images during sunitinib therapy.** Transverse enhanced MR T1-weighted images **(A**, **B)** and MR diffusion weighted images (DWIs) for estimated apparent diffusion coefficient (ADC) **(C**, **D)** at baseline and after 2-week of sunitinib therapy in the same 47-year-old man with HCC (arrows). After two weeks of treatment with sunitinib, there was an increase in ADC value from 0.86 × 10-3 mm2/s to 0.94 × 10-3 mm2/s.

The tumor parameters at baseline and 2-week post- sunitinib therapy are shown in Table 1. There was minimal change in median tumor burden based on RECIST (from 10.00 cm to 11.10 cm, *P* = 0.197) and mRECIST (from 9.94 cm to 10.11 cm, *P* = 0.96) and median tumor thrombus diameter (from 28.67 mm to 29.81 mm, *P* = 0.365). There was an increase in median tumor ADC value from 0.88 × 10^-3^ mm^2^/s to 0.98 × 10^-3^ mm^2^/s (*P* < 0.0001) and in median tumor thrombus ADC value from 0.89 × 10^-3^ mm^2^/s to 1.02 × 10^-3^ mm^2^/s (*P* = 0.035). The Ktrans change of HCC in MRP was more substantial with a decrease from median baseline value of 2.15 min^−1^ to 2-week post-treatment value of 0.94 min^−1^ (*P* < 0.0001). The HCC also showed relatively higher median Kep at baseline (4.15 min^−1^) and a significant decrease compared with post-treatment value (1.44 min^−1^) (*P* < 0.0001). The tumor thrombus displayed similar diffusion and perfusion features as their primary HCC at baseline and after treatment. The examples of Ktrans and ADC changes in an indexed HCC lesion after sunitinib administration are illustrated in Figures 1 and 2.

**Table 1:** Tumor and tumor thrombus size, DWI and MRP parameters at baseline and post antiangiogenic treatment

**Parameters**		**Baseline (Median, Range)**	**Post-sunitinib (Median, Range)**	***P *value**
**SIZE**				
***RECIST***		10.00, 2.30 ~ 17.00	11.10, 1.30 ~ 25.00	0.197
***m RECIST***		9.94, 2.15 ~ 19.56	10.11, 2.11 ~ 18.55	0.963
***Tumor thrombus(mm)***		28.67, 21.87 ~ 36.63	29.81, 21.98 ~ 37.73	0.365
**DWI**				
***HCC***	*ADC (×10*^*-3*^*mm*^*2*^*/s)*	0.88, 0.58 ~ 1.09	0.98, 0.75 ~ 1.21	<0.0001
***Tumor thrombus***	*ADC (×10*^*-3*^*mm*^*2*^*/s)*	0.89, 0.60 ~ 1.14	1.02, 0.78 ~ 1.26	0.035
**MRP**				
***HCC***	*Ktrans (min*^*−1*^*)*	2.15, 0.90 ~ 4.81	0.94, 0.57 ~ 2.30	<0.0001
	*Kep (min*^*−1*^*)*	2.61, 1.00 ~ 41.75	1.22, 0.66 ~ 2.64	<0.0001
	*EVF*	0.926, 0.34 ~ 1.00	0.81, 0.65 ~ 0.97	0.079
***Tumor thrombus***	*Ktrans (min*^*−1*^*)*	1.89, 0.93 ~ 2.99	0.99, 0.37 ~ 1.61	0.110
	*Kep (min*^*−1*^*)*	2.37, 1.05 ~ 4.03	1.49, 0.61 ~ 2.37	0.190
	*EVF*	0.83, 0.70 ~ 0.98	0.66, 0.56 ~ 0.77	0.055

Abbreviations: *Ktrans* Transfer constant; *Kep* Rate constant; *EVF* Extracellular volume fraction; *ADC* Apparent diffusion coefficient.

### Correlation of baseline and percent changes in DWI and MRP parameters with clinical outcome

For correlation with clinical outcome, patients were divided into two groups: responders (RR) and nonresponders (NR) after completion of 2-cycles (12 weeks) of sunitinib therapy in 23 patients. 16 (69.6%) were classified as RR (PR + SD) and the remaining 7 as NR (PD, 30.4%). In the RR, median baseline RECIST and mRECIST derived tumor burden was 9.50 cm and 10.14 cm with a median percent change of 1.15% and 1.94%, whereas in the NR the median tumor burden was 10.30 cm with a median percent change of 33.98% (baseline P = 0.255 and percent change P = 0.0002) for RECIST and 6.12 cm with a median percent change of 0.49% (baseline P = 0.290 and percent change P = 0.360). The median baseline tumor thrombus diameter in RR and NR was 26.42 mm and 31.48 mm (*P* = 0.110) without any significant change at the end of sunitinib treatment (−10.22% and 7.72%, *P* = 0.260). Likewise, although the median baseline ADC value was relatively higher in NR, there was no significant difference in the median baseline ADC value as well as the median percent change of HCC and tumor thrombus between RR and NR.

In contrast, Ktrans in RR showed significantly larger median percent change (−54.98%) than NR (−17.83%) (*P* = 0.014). Similarly, Kep in RR showed significantly larger median percent change (−42.06%) than NR (−24.21%) (*P* = 0.048). Moreover, the median percent Ktrans change of tumor thrombus in RR (−55.00%) was also larger than that in NR (−20.04%) (*P* = 0.006). The median baseline as well as the median percent changes of EVF values in HCC and tumor thrombus didn’t show any significant difference between RR and NR (Table 2).

**Table 2:** Correlation of DWI and MRP parameters with clinical outcome in HCC and tumor thrombus

			**Baseline**		**Percent changes (%)**		
**Parameters**		**SD or PR**	**PD**	***P *value**	**SD or PR**	**PD**	***P *value**
		**(Median, Range)**	**(Median, Range)**		**(Median, Range)**	**(Median, Range)**	
**RECIST**							
***HCC (cm)***		9.50, 2.30 ~ 17.00	10.30, 4.30 ~ 10.3	0.255	1.15, –43.48 ~ 8.46	33.98, 20.71 ~ 58.23	0.0002
***Tumor thrombus(mm)***		26.42, 22.14 ~ 30.70	31.48, 24.65 ~ 38.34	0.110	−10.22, –25.62 ~ 10.26	7.72, –28.52 ~ 42.92	0.260
**mRECIST**							
***HCC (cm)***		10.14, 2.15 ~ 19.56	6.12, 2.24 ~ 15.86	0.290	1.94, –19.80 ~ 15.86	0.49, –19.84 ~ 6.70	0.360
**DWI**							
***HCC***	*ADC (×10*^*-3*^*mm*^*2*^*/s)*	0.86, 0.58 ~ 0.99	0.93, 0.8 ~ 1.09	0.15	22.42, –8.99 ~ 51.72	22.22, –12.84 ~ 25.81	0.300
***Tumor thrombus***	*ADC (×10*^*-3*^*mm*^*2*^*/s)*	0.66, 0.60 ~ 0.73	0.96, 0.79 ~ 1.14	0.290	13.34, –13.28 ~ 39.94	20.32, –62.33 ~ 114.23	0.140
**MRP**							
***HCC***	*Ktrans (min*^*−1*^*)*	2.35, 0.90 ~ 4.81	1.29, 1.00 ~ 2.80	0.402	−54.98, –83.37 ~ –10.00	−17.83, –72.09 ~ –6.00	0.014
	*Kep (min*^*−1*^*)*	4.25, 1.00 ~ 41.75	1.54, 1.00 ~ 3.01	0.18	−54.73,– 96.32 ~ −5.04	−12.19, –70.17 ~ 28.04	0.048
	*EVF*	0.83, 0.34 ~ 1.00	0.95, 0.84 ~ 1.00	0.065	−2.38, –47.50 ~ 61.76	-6.45, –27.37 ~ –2.38	0.331
***Tumor thrombus***	*Ktrans (min*^*−1*^*)*	1.96, 0.93 ~ 2.99	1.73, 1.05 ~ 2.41	0.350	−55.00, –72.65 ~ −47.35	−20.04, –63.23 ~ 24.14	0.006
	*Kep (min*^*−1*^*)*	2.54, 1.05 ~ 4.03	1.97, 1.18 ~ 2.76	0.230	−45.53, –67.42 ~ −23.64	−30.58, –80.01 ~ 18.98	0.090
	*EVF*	0.81, 0.70 ~ 0.92	0.89, 0.82 ~ 0.98	0.110	−15.70, –31.95 ~ 0.56	−29.65, –38.12 ~ −21.07	0.060

Abbreviations: *Ktrans* Transfer constant; *Kep* Rate constant; *EVF* Extracellular volume fraction; *ADC* Apparent diffusion coefficient; *SD* Partial disease; *PR* Partial response; *PD* Progressive disease.

### Correlation of baseline and percent changes in DWI and MRP parameters at 2-weeks with PFS

We then examined the correlation of baseline and percent changes in DWI and MRP parameters with PFS. The median PFS time of study cohort was 4.6 months (95% confidence interval [CI], 3.8 ~ 8.9 months) and the updated overall survival time was 9.9 months (95% CI, 7.5 ~ 14.1 months). The PFS rate at 6 months was 39.1%.

The median baseline ADC value was relatively higher in patients with PFS ≤ 6 months but not statistically different from the median baseline ADC value as well as the median percent change of HCC and tumor thrombus between these two groups.

In contrast, the median baseline Ktrans in patients with PFS > 6 months (2.80 min^−1^) was significantly higher than that in those with PFS ≤ 6 months (1.70 min^−1^) (*P* = 0.043). Moreover, the median percent change of tumor thrombus in patients with PFS > 6 months (−58.48%) was also larger than those with PFS ≤ 6 months (−23.11%) (*P* = 0.005). The median percent changes of EVF value in HCC showed a significant correlation with PFS (*P* = 0.022) (Table 3).

**Table 3:** Correlation of DWI and MRP parameters with PFS in HCC and tumor thrombus

			**Baseline**		**Percent changes (%)**		
**Parameters**		**PFS>6 months**	**PFS6 ≤ months**	***P *value**	**PFS>6 months**	**PFS6 ≤ months**	***P *value**
		**(Median, Range)**	**(Median, Range)**		**(Median, Range)**	**(Median, Range)**	
**RECIST**							
***HCC (cm)***		10.00, 5.20 ~ 14.00	10.00, 2.30 ~ 17.00	0.682	3.57, –36.54 ~ 35.00	7.60, –43.48 ~ 58.23	0.875
***Tumor thrombus(mm)***		23.87, 22.14 ~ 28.50	30.79, 24.65 ~ 38.34	0.114	−15.23, –25.62 ~ 10.26	9.72, –28.52 ~ 42.92	0.019
**mRECIST**							
***HCC (cm)***		10.70, 2.15 ~ 19.56	9.66, 2.24 ~ 15.86	0.207	−0.88, –11.16 ~ 11.00	1.89, –19.84 ~ 15.86	0.517
**DWI**							
***HCC***	*ADC (×10*^*-3*^*mm*^*2*^*/s)*	0.83, 0.65 ~ 1.01	0.90, 0.69 ~ 1.09	0.570	19.48, –12.84 ~ 40.00	22.22, –8.99 ~ 51.72	0.825
***Tumor thrombus***	*ADC (×10*^*-3*^*mm*^*2*^*/s)*	0.74, 0.60 ~ 1.03	0.93, 0.79 ~ 1.14	0.085	10.38, –13.28 ~ 39.94	21.11, –62.33 ~ 114.23	0.315
**MRP**							
***HCC***	*Ktrans (min*^*−1*^*)*	2.80, 1.00 ~ 4.81	1.70, 0.90 ~ 4.1	0.043	−52.27, –83.37 ~ −7.00	−41.25, –75.61 ~ -6.00	0.467
	*Kep (min*^*−1*^*)*	3.01, 1.00 ~ 41.75	2.79, 1.27 ~ 4.36	0.100	−49.58, – 96.32 ~ 28.04	−46.33, –77.05 ~ -1.67	0.914
	*EVF*	0.93, 0.57 ~ 1.00	0.88, 0.34 ~ 1.00	0.590	−8.77, –47.50 ~ 20.55	–2.38, –30.12 ~ 61.76	0.022
***Tumor thrombus***	*Ktrans (min*^*−1*^*)*	1.65, 0.93 ~ 2.33	1.98, 1.05 ~ 2.99	0.374	−58.48, –72.65 ~ −47.35	−23.11, –63.23 ~ 24.14	0.005
	*Kep (min*^*−1*^*)*	2.43, 1.32 ~ 4.03	1.95, 1.05 ~ 2.76	0.220	−42.53, –67.42 ~ −23.64	−34.58, –80.01 ~ 18.98	0.135
	*EVF*	0.78, 0.70 ~ 0.92	0.88, 0.82 ~ 0.98	0.120	−11.30, –31.95 ~ 0.56	−22.43, –38.12 ~ −19.75	0.331

Abbreviations: *Ktrans* Transfer constant; *Kep* Rate constant; *EVF* Extracellular volume fraction; *ADC* Apparent diffusion coefficient.

### Changes in vascular permeability measured by MRI and circulating biomarkers

The changes in circulating biomarkers at 2 weeks after sunitinib treatment have been reported elsewhere [10]. When we compared the change in MRI parameters with the change in plasma angiogenic and inflammatory cytokines we found a significant correlation between decrease in sVEGFR2 or TNF-α and a drop in Kep (*P* = 0.030 and *P* = 0.035 respectively) and a similar correlation for the decrease in Ktrans (*P* = 0.044 and *P* = 0.051 respectively). There was no other association between the changes in MRI parameters and circulating biomarkers at this time-point (Table 4).

**Table 4:** **Correlation between changes in MRI parameters and plasma biomarkers after sunitinib treatment (Kendall’s *τ *correlation coefficients)**

**Kendall’s τ**	**Ktrans**	**Kep**	***EVF***	***ADC***
**VEGF**	0.00*	0.11	0.03	0.24
*P-*value	1.00	0.49	0.86	0.13
**PlGF**	0.00	0.10	−0.06	0.06
*P-*value	0.74	0.53	0.72	0.72
**sVEGFR1**	0.22	0.18	−0.16	−0.16
*P-*value	0.16	0.26	0.30	0.30
**sVEGFR2**	***0.33***	***0.35***	−0.05	−0.19
*P-*value	***0.044***	***0.030***	0.77	0.24
**sVEGFR3**	0.00	−0.09	0.24	−0.23
*P-*value	1.00	0.62	0.21	0.22
**VEGF-C**	0.17	0.17	0.02	−0.01
*P-*value	0.28	0.29	0.90	0.95
**bFGF**	0.17	0.15	0.01	−0.06
*P-*value	0.28	0.45	0.86	0.72
**TNF-α**	*0.32*	***0.34***	0.01	−0.17
*P-*value	*0.051*	***0.030***	0.97	0.30
**SDF1α**	0.02	0.14	0.12	0.26
*P-*value	0.90	0.38	0.47	0.10
**IL-1β**	0.16	0.14	0.16	−0.02
*P-*value	0.33	0.38	0.31	0.90
**IL-6**	0.01	−0.04	−0.04	−0.13
*P-*value	0.95	0.82	0.82	0.44
**IL-8**	0.10	0.08	0.04	−0.21
*P-*value	0.56	0.63	0.82	0.19
**Sol c-KIT**	0.20	0.12	−0.01	0.01
*P-*value	0.20	0.45	0.95	0.95

*Positive values of Kendall’s *τ* indicate a direct correlation between changes after treatment (relative to baseline) in imaging biomarkers and plasma biomarkers.

## Discussion

The RECIST based change in tumor burden following treatment with chemotherapy is a widely accepted imaging surrogate for assessing treatment outcome in oncologic clinical trials. Its ease of use, quantization and reproducibility has been the major attribute for its success. Due to deficiencies in RECIST for evaluating treatment efficacy in HCC, the criteria have been modified (mRECIST) to include arterial phase enhancement of the lesion [16]. However, novel targeted antiangiogenic approaches may induce necrosis and stabilize tumor growth rather than tumor regression, which makes the early response evaluation challenging. In this context, there has been an increase in the utilization of MRP in HCC, including for monitoring early therapeutic effects after a few days/weeks of antiangiogenic treatment [12,26,27]. An advantage of the MRP technique is that it can be incorporated into routine conventional MRI providing physiological information. Moreover, MRP coupled with powerful and user-friendly software packages can offer excellent contrast resolution, more coverage, repeated examinations and continuous sampling of data for more than four minutes, which allows the assessment of washout without exposure to ionizing radiation.

In this study, we observed that the MRP derived HCC parameters (Ktrans and Kep) were more sensitive imaging biomarkers than ADC value, RECIST, and mRECIST for monitoring early antiangiogenic treatment effects. Ktrans (wash-in rate) describes the leakage rate of the contrast medium. For blood vessels where leakage is rapid, that is, when extraction fraction during the first pass of the contrast agent is high, perfusion will determine contrast agent distribution and Ktrans approximates to tissue blood flow per unit volume [25]. Whereas, Kep measures the rate of contrast agent diffusion back into the vasculature (wash-out rate) from where it is excreted [25]. Higher baseline Ktrans value and more substantial drop in Ktrans and Kep at 2 weeks after therapy correlated with better clinical outcome or PFS. Our results support the hypothesis that after antiangiogenic therapy, the changes in tumor perfusion precede the change in tumor size, which make the MRP parameters more sensitive for monitoring early antiangiogenic effects compared with tumor burden measurements as defined by RECIST or mRECIST in advanced HCC. The reduced tumor vessel permeability as estimated by MRP indicated a direct effect on HCC microvasculature that might be associated with clinical benefit after sunitinib therapy. Similar observations have been reported by de Langen AJ et al. in patients with advanced non-small cell lung cancer treated with bevacizumab and erlotinib [28]. In advanced HCC, DCE-MRI demonstrated reduction in Ktrans during antiangiogenic treatment and the change of Ktrans during treatment was related to better PFS and OS in clinical trials of sorafenib [26,27]. The change of Ktrans may reflect the underlying tumor permeability changes induced by antiangiogenic therapy. This suggests that control of vessel leakiness may be a determinant of HCC response to sunitinib [28,29].

In addition, we found the higher baseline of Ktrans can even relate with longer PFS. Similar studies on the predictive value of tumor perfusion parameters have also been reported. In cervical cancer, the MRP parameters quantifying permeability status can provide very early prediction of tumor control and disease-free-survival [30]. The MRP parameters such as Ktrans depend on vascular permeability and are being considered as imaging biomarker because they can detect functional changes in tumor vasculature after treatment with anti-VEGF agents [31]. The increased concentration gradient across the endothelial membrane, the larger surface area of the vascular endothelium to which they are exposed, the higher endothelial permeability, the loss of cell membrane integrity and higher cellular density can all contribute to the relatively higher baseline permeability values in responders and patients with longer PFS [25,32,33].

Increase in ADC in response to sunitinib therapy is consistent with the observations made by other investigators on ADC increase in liver tumors to other non-surgical treatments and is believed to result from loss of cell membrane integrity or necrosis [34,35]. However, the median baseline value and the percent change of ADC did not correlate with the clinical outcome and PFS. It is possibly due to higher biologic aggressiveness and presence of tumor necrosis. Poor perfusion is known to impede drug delivery and induce hypoxic and acidic environment, which diminishes the effectiveness of antiangiogenic therapy [36,37]. In addition, following antiangiogenic therapy, improvement of tumor perfusion, edema and inflammation may be the dominant factor to influence the ADC values instead of necrosis and loss of cell membrane integrity that often follows much later in the course of system chemotherapy [38]. This potentially explains mild changes in ADC in comparison to more significant changes in Ktrans and Kep.

Interestingly, we also found that tumor thrombus showed high baseline values and substantial reduction in perfusion parameters following sunitinib treatment almost similar to the response in the primary tumor. The accurate differentiation of bland from tumor thrombus is crucial for patient treatment. Although a neoplastic thrombus can be discriminated from a clot in most cases by CE-MRI alone, characterization of small thrombi or a peripheral one can be difficult on conventional MR alone. Our results support the tissue characterization benefits of MRP parameters as well as DWI, which could potentially be applied in differentiating tumor thrombus from bland thrombus [23]. Moreover, tumor thrombus could be the only visible evidence of measurable disease and might be used for response evaluation.

Signaling through VEGFR2 in endothelial cells is critical in VEGF-induced vascular leakiness [39,40], and a decrease in circulating sVEGFR2 has been consistently seen with all agents that block this pathway [10,41,42]. Similarly, TNF-α—a pro-inflammatory cytokine—is known to increase vascular permeability [43]. The preliminary finding that the changes in MRI parameters are associated with the changes in circulating sVEGFR2 and TNF-α suggests that the rapid drop in vessel leakiness in HCC after sunitinib treatment may potentially occur by direct blockade of VEGF/VEGFR2 signaling or indirectly by reduction of TNF-α. In this study, Kep showed significant correlations with both VEGFR2 and TNF-α, whereas Ktrans showed a significant correlation with only VEGFR2. There was a trend for correlation between Ktrans and TNF-α, but it did not reach statistical significance, which may be due to the small sample size of this study. These associations should be tested in larger prospective studies.

It should be noted that our study has a few limitations. First, the sample size is relatively small and the predictive value of Ktrans and Kep remains to be validated in larger prospective studies. In addition, the diagnosis of tumor invasion in the portal vein was based on well-established imaging criteria of portal vein expansion and appreciable enhancement in the thrombus and not on histopathologic sampling.

## Conclusions

Our experience from this phase II study suggests that following antiangiogenic therapy in advanced HCC, imaging changes are detectable within 2 weeks on DWI and MRP-derived parameters. Moreover, larger percent drops in Ktrans and EFV – but not the changes in tumor burden – correlated with longer PFS, which suggests that they are potentially superior imaging biomarkers of response for antiangiogenic therapy in HCC. These results may be specific to the method of analysis and the software employed in this study and warrant validation in future studies. However, these data indicate that MRI-based evaluations of tumor diffusion and perfusion and circulating biomarker evaluation will not only provide a better mechanistic understanding of the effects of antiangiogenic therapies, but will also facilitate tumor response assessment.

## Abbreviations

ADC: Apparent diffusion coefficient; DWI: Diffusion-weighted imaging; EVF: Extracellular volume fraction; HCC: Hepatocellular carcinoma; Ktrans: Transfer constant; Kep: Redistribution rate constant; MRI: Magnetic resonance imaging; MRP: MR perfusion; PFS: Progression free survival; RECIST: Response Evaluation, Criteria In Solid Tumors; ROI: Region of interest; VEGF: Vascular endothelial growth factor.

## Competing interests

Andrew X Zhu, M.D. served as a consultant for Pfizer. Rakesh K. Jain received research grants from Dyax, MedImmune and Roche; received consultant fees from Dyax, Enlight, Noxxon, and Zyngenia; owns equity in Enlight, SynDevRx, and XTuit; and serves on the Board of Directors of XTuit and Boards of Trustees of H&Q Healthcare Investors and H&Q Life Sciences Investors. No reagents or funds from these organizations were used in this study. All other authors declare no conflicts.

## Authors’ contributions

DVS participated in data collection, data analysis, prepared the manuscript for submission and manuscript preparation. TJ participated in data collection, data analysis and prepared the manuscript for submission. KH prepared the manuscript for submission, participated in data collection, data analysis and manuscript revision. DGD participated in data collection, data analysis and manuscript preparation. OAC participated in data collection, data analysis and manuscript preparation. AM participated in data collection, data analysis and manuscript revision. RKJ participated in treatment planning, data collection, data analysis and manuscript preparation. AXZ participated in treatment planning and manuscript preparation. All authors have read and approved the final manuscript.
